# Incidence of Post-extubation Stridor in Infants With Cuffed vs. Uncuffed Endotracheal Tube: A Retrospective Cohort Analysis

**DOI:** 10.3389/fped.2022.864766

**Published:** 2022-05-11

**Authors:** Katharina Bibl, Lena Pracher, Erik Küng, Michael Wagner, Imme Roesner, Angelika Berger, Michael Hermon, Tobias Werther

**Affiliations:** ^1^Division of Neonatology, Pediatric Intensive Care and Neuropediatrics, Department of Pediatrics and Adolescent Medicine, Comprehensive Center for Pediatrics, Medical University of Vienna, Vienna, Austria; ^2^Department of Otorhinolaryngology, Head and Neck Surgery, Medical University of Vienna, Vienna, Austria

**Keywords:** intubation, infant, endotracheal tube, post-extubation stridor, subglottic stenosis

## Abstract

**Background:**

Endotracheal intubation is a common procedure in Neonatal Intensive Care. While cuffed endotracheal tubes (ETT) are the standard of care in adults and children, their use in infants is controversial. The aim of this study was to compare the incidence of post-extubation stridor between uncuffed and cuffed ETTs in infants. We further evaluated the safety of cuffed ETTs in infants with a bodyweight between 2 and 3 kg and performed baseline analysis on development of subglottic stenosis.

**Methods:**

In this retrospective study, we screened all infants admitted to two NICUs of the Medical University of Vienna between 2012 and 2019.The study cohort was screened twice: In the first screening we selected all infants who underwent the first intubation when attaining a bodyweight >2 kg (but <6 kg) to analyze the incidence of post-extubation stridor and only considered the first intubation of each included infant. Post-extubation stridor was defined as the administration of either epinephrine aerosol or any corticosteroid within 6 h post-extubation. In the second screening we searched for all infants diagnosed with acquired severe subglottic stenosis during the study period regardless their bodyweight and numbers of intubations.

**Results:**

A total of 389 infants received at least one intubation during the study period. After excluding infants who underwent the first intubation below a bodyweight of 2 kg, 271 infants remained for final analysis with an average gestational age of 38.7 weeks at the time of intubation. Among those, 92 (33.9%) were intubated with a cuffed and 179 (66.1%) with an uncuffed ETT. Seven infants (2.6%) developed a clinically significant stridor: five of those were intubated with a cuffed and two with an uncuffed ETT (71.4 vs. 28.6%, *p* = 0.053). All of them had a bodyweight >3 kg at the time of intubation. Infants who developed subglottic stenosis were more often intubated with an uncuffed ETT.

**Conclusion:**

In this study, no difference in the incidence of post-extubation stridor between cuffed and uncuffed ETTs in infants with a bodyweight from 2 to 6 kg could be found. The use of uncuffed ETTs does not exhibit higher risk for the acquired subglottic stenosis in this cohort.

## Introduction

Endotracheal intubation is a frequently performed procedure at Neonatal Intensive Care Units (NICUs). To avoid airway injuries and long-term complications in infants, choosing the appropriate endotracheal tube (ETT) in size and type is of high importance (1). While cuffed ETTs are the standard of care in adults and children beyond infancy, their use in infants is still controversial (2). Due to concerns about cuff-related trauma, infants are mostly intubated using uncuffed ETTs (3, 4) although invasive ventilation with a cuffed ETT brings along several advantages. The use of cuffed ETTs results in decreased risk of aspiration (5, 6), possibility to adjust the fit of cuffed ETTs by changing the cuff volume (7), fewer replacements of poorly fitting tubes (8–10) and a smaller percentage of significant air leak (9, 11). A considerable drawback of cuffed ETTs in infants is the increased airway resistance due to the smaller internal diameter (12, 13).

As for today, there is no conclusive data on airway damage in infants resulting from cuffed ETTs (14). Tube insertion and maintenance can cause laryngeal edema. One commonly accepted clinical marker for post-extubation laryngeal edema is the development of inspiratory stridor after extubation (15). It is specific in detecting moderate to severe injury in the upper airway; however, it exhibits low sensitivity (16). As far as the pathogenesis of many of the injuries is concerned, ischemic necrosis between the ETT and the laryngeal surface is often an important factor if the pressure of the ETT exceeds the mucosal capillary perfusion pressure, which is estimated to be 20–30 cm H2O (17–19).

Several recent studies have investigated the use of cuffed ETTs in children. None of them found any difference in the incidence of stridor or croup between cuffed and uncuffed ETTs (8, 10, 20). Weiss et al. (10) emphasized the importance of introducing a policy of appropriate placement of cuffed ETTs and maintenance including regular monitoring of the cuff pressure.

Acquired subglottic stenosis (SGS) is a rare long-term complication associated with endotracheal intubation and is diagnosed by laryngeal endoscopy. The incidence varies widely and is believed to range from 0.3 to over 11.0% (21–23). Therapy options include endoscopic dilatation, tracheostomy or surgical airway reconstruction (24). In 2018, Greaney et al. did not find any correlation between the use of cuffed ETTs and the development of SGS in a retrospective pediatric cohort analysis (25).

The aim of this retrospective study was to compare the incidence of post-extubation stridor between uncuffed vs. cuffed ETTs in infants during an observational period of seven years. We further evaluated the safety of cuffed ETTs in infants with a body weight between 2 and 3 kg and performed a baseline analysis of all infants at our NICUs, who suffered from acquired SGS. We hypothesized, that the use of cuffed ETTs was safe, even in infants below 3 kg, and did not lead to higher rates of post-extubation stridor.

## Methods

We performed a retrospective chart analysis. All infants admitted to the two level III NICUs at the Medical University of Vienna between January 1st 2012 and March 31st 2019 were screened. All infants suffering from SGS during their stay were admitted to these units, respectively. Approval was given by the Institutional Review Board of the Medical University of Vienna (N. 1581/2019).

Following our local policy, ETTs with cuff (Kimberly-Clarke Microcuff ID 3.0 for infants with more than 3 kg body weight and Rüsch Super Safety ID 2.5 for infants with a bodyweight between 2 kg and 3 kg) were only used when surgical interventions were planned.

Data was accessed and extracted from the local patient data management system chart (ICCA, IntelliSpace Critical Care and Anesthesia, Phillips, NL).

The selection was limited to those infants who had been intubated with an ETT during their stay, irrespective of whether intubation had been performed at our NICU or elsewhere (e.g., operating room, external institution). The cohort was further restricted to infants with a body weight ranging from 2 to 6 kg at the time of ETT insertion. We excluded all infants with congenital airway malformations. Infants whose ETT was changed or those who deceased prior to extubation, were also excluded. Finally, the study-cohort was divided into two groups: infants intubated with a cuffed ETT (cuffed group) and infants intubated with an uncuffed ETT (uncuffed group).

To reduce any bias due to repeated intubation procedures, we only considered the first intubation and extubation of every infants. Therefore, the number of intubations is equivalent to the number of infants.

The primary outcome was defined as the incidence of post-extubation stridor. As stridor is not explicitly documented in our medical records, we defined the occurrence of post-extubation stridor whenever either nebulized epinephrine or any type of intravenous, oral, rectal or nebulized corticosteroid with the indication for upper airway obstruction, or both, had been administered within the first 6 h post-extubation (2). As data on the use of cuffed ETTs in infants below 3 kg are rare, we highlighted our findings concerning post-extubation stridor for infants below a bodyweight of 3 kg in a stand-alone paragraph.

We analyzed baseline and intubation characteristics of the two study groups including sex, gestational age at birth, chronological age (days of life and gestational age), and bodyweight at the time of intubation.

We collected data regarding the place of intubation (NICU, operating room, external institution), days of invasive ventilation, intubation route (nasal/oral), diameter and insertion depth of the ETT, maximal cuff pressure, and the administration of intravenous corticosteroids prior to planned extubation.

Infants from our institution, who needed surgical procedures because of severe SGS, were always (re)-admitted to our units throughout the study episode. This gave us the opportunity to cross-match our initial study cohort with all infants diagnosed with severe acquired SGS. To get an overview of the total incidence of intubation acquired SGS in all our patients, we did not limit the patient cohort for this part of the study to a certain bodyweight. To allow a broader view on the causes of SGS in our patients, apart from the type of ETT (cuffed vs. uncuffed), we also included patients below 2 kg. For these infants, we also collected data on gestational age, body weight, number of intubations, and findings in laryngeal endoscopy.

Statistical analysis was performed using SPSS (IBM SPSS statistics, Version 26, IBM Corporation, New York, USA), and R *(R Foundation for Statistical Computing, Vienna, Austria, version 4.1.1)*. Baseline characteristics were summarized using absolute and relative frequencies. Metric data is reported as mean (SD) or median (IQR). For baseline characteristics, we furthermore used a chi-square or Fisher's exact test (small sample size) for categorical values and Mann-Whitney U test for not normally distributed metric variables. If metric data was distributed normally, we used a *t*-test. Statistical comparison of post-extubation stridor between the study groups was performed using a Fisher's Exact Test. Calculation of Odds Ratio and confidence interval was used to analyze risk for post-extubation stridor necessitating nebulized epinephrine and/or corticosteroids between the two groups (cuffed/uncuffed). Multivariate logistic regression was performed using the “glm” function with “binomial family” in “R” (26). The level of significance was set at *p* < 0.05 (two-tailed).

## Results

**Figure 1:**
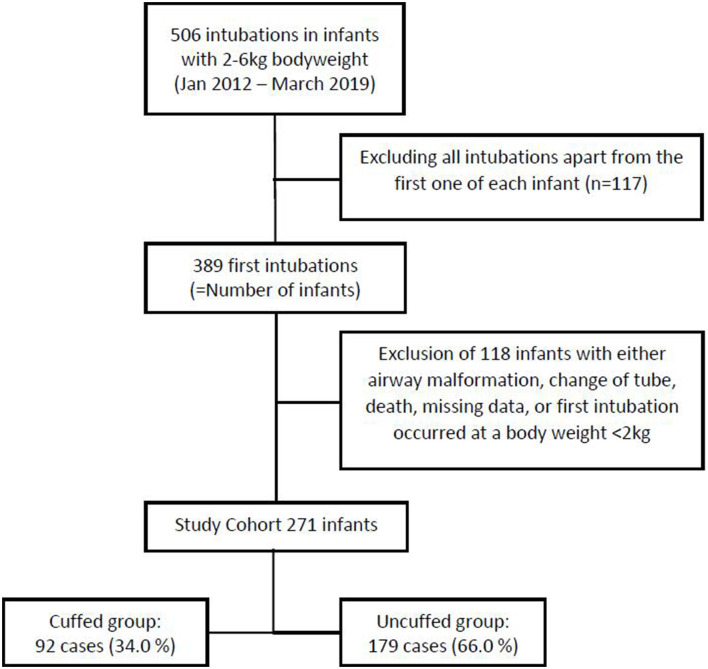
Study cohort after applying exclusion criteria.

From January 1st 2012 to March 31st 2019, there were 4979 admissions to our NICUs. Among those, 1,098 underwent invasive mechanical ventilation via endotracheal intubation. Considering only the first endotracheal intubation that occurred at a bodyweight ranging from 2 to 6 kg, we identified 389 intubations, i.e., infants. After excluding 118 infants due to missing entries in the electronic patient chart, death prior to extubation, ETT exchange, congenital malformations of the airway, and those who had been intubated before they reached a bodyweight of 2 kg, 271 infants met the inclusion criteria (Figure 1).

### Baseline Characteristics

**Figure 2:**
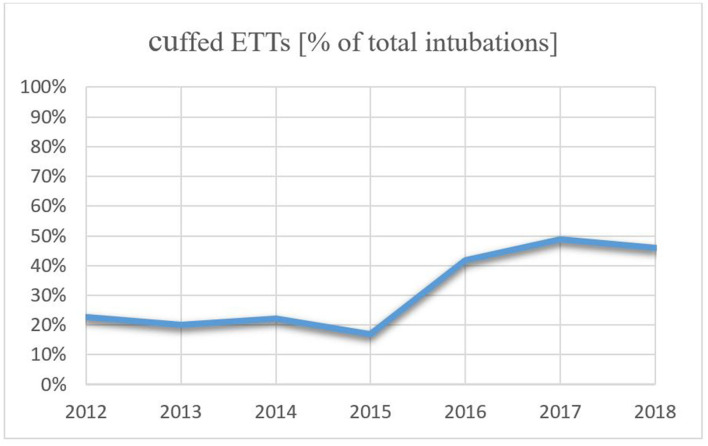
Trend of use in cuffed ETTs from 2012 to 2018.

Within this study cohort, 92 infants (34.0%) were intubated with a cuffed ETT, while an uncuffed ETT was used in 179 infants (66.0%). The use of cuffed ETTs per year increased over the study period from 22.73% in 2012 to 45.90% in 2018, which is an increase of 23.17% (Figure 2). Patient characteristics are presented in Table 1. Infants intubated with a cuffed ETT showed a significant shorter time of invasive ventilation compared to the uncuffed ETT (1 day vs. 3 days; *p* < 0.001). The route of intubation was mostly nasal in both groups (90.2% cuffed group vs. 97.8% uncuffed group) with a slightly significant difference in the insertion depth (11.0 cm in the cuffed group vs. 10.5 cm in the uncuffed group; *p* = 0.04). The median inner diameter of the cuffed ETT was significantly smaller than for the uncuffed ETT (3.0 mm vs. 3.5 mm, *p* < 0.001). Cuff pressure was 12.0 cm H_2_O on average. ETTs with cuff were most often inserted in the operating room (Table 1). Corticosteroids prior to extubation were administered more often in the cuffed group (14.1 vs. 2.2%; *p* < 0.001) (Table 1).

**Table 1:** Baseline patient and intubation characteristics.

**Variable**	**Cuffed Group**	**Uncuffed Group**	**Total**	*p*-value^***^
Patients, *n*	92	179	271	
Male, *n* (%)^*^	48 (52.0)	97 (54.0)	145 (54.0)	0.75
Gestational age at birth, median (25., 75. perc.), [weeks +days]^**^	37+5 (36+1, 39+0)	38+0 (35+5, 40+1)	38+0 (35+6, 39+6)	0.34
Days of life at intubation, median (25., 75. perc.), [days]^**^	8 (3-24)	2 (1-7)	3 (1-12)	<0.001
Bodyweight at intubation, median (25., 75. perc.), [kg]^**^	3.2 (2.78, 3.65)	3.1 (2.56, 3.66)	3.1 (2.62, 3.67)	0.23
location of intubation*				
Ward, *n* (%)	40 (44.0)	147 (82.0)	187 (69.0)	<0.001
Operating room, *n* (%)	52 (57.0)	4 (2.0)	56 (21.0)	<0.001
External, *n* (%)	0 (0)	28 (16.0)	28 (10.0)	<0.001
Duration of invasive ventilation, median (25.0, 75.0 Perc.), [days]^**^	1 (1, 2)	3 (1, 6)	2 (1, 4)	<0.001
Nasal route of intubation, *n* (%)^*^	83 (90.0)	175 (98.0)	258 (95.0)	0.012
Insertion depth for nasal intubation, median (25.0, 75.0 Perc.), [cm]^**^	11.0 (10.0, 11.5)	10.5 (10.0, 11.0)	10.5 (10.0, 11.0)	0.037
Insertion depth for oral intubation, median (25.0, 75.0 Perc.), [cm]^**^	9.0 (8.5, 10.0)	9 (9.0, 9.0)	9 (9.0, 9.5)	0.4
Diameter of tube, median (25.0, 75. Perc.), [mm]^**^	3 (3.0, 3.0)	3.5 (3.5, 3.5)	3.5 (3.0, 3.5)	<0.001
Maximal cuff pressure, median (25.0, 75.0 Perc.), [cmh2o]^**^	12 (10, 20)	-	-	-
Administration of corticosteroid before extubation, *n* (%)^*^	13 (14.1)	4 (2.2)	17 (6.3)	<0.001

* *Results are shown in absolute frequency and frequency in percentage*.

** *Results are shown in median and interquartile range*.

*** *Chi-square test (big sample size) and Fisher's exact test (small sample size) for categorical variables; Mann-Whitney-U Test for metric variables if data was not normally distributed and a t-test if data was normally distributed*.

### Primary Outcome

In total, seven infants (2.6%) developed stridor after extubation. Five of these infants were intubated with a cuffed ETT while two infants were intubated with an uncuffed ETT (5.4 vs. 1.1%, *p* = 0.053). The risk of post-extubation stridor was not significantly higher for cuffed than for uncuffed ETTs (OR 4.86, 95 %-CI 0.93- 25.56) (Table 2). Multivariate logistic regression identified only corticosteroids before extubation to be significantly associated with post-extubation stridor (*p* = 0.00015), whereas weight at intubation (*p* = 0.085), duration of intubation (*p* = 0.75), post menstrual age at intubation (*p* = 0.99), day of life at intubation (*p* = 0.45) and cuffed endotracheal tube (*p* = 0.7) were not significantly associated with the development of post-extubation stridor.

**Table 2:** Post-extubation stridor in patients being intubated with cuffed or uncuffed endotracheal tubes.

	**Cuffed group (*n* = 92)**	**Uncuffed group (*n* = 179)**	**Total (*n* = 271)**	*p*-value^*^	**OR (95%-CI)**
Stridor, *n* (%)	5 (5.4)	2 (1.1)	7 (2.6)	0.053	4.86 (0.93–25.56)

* *Fisher's Exact Test*.

### Intubation With a Cuffed Endotracheal Tube Below 3 kg

In total, 35 out of 111 infants with a bodyweight below 3 kg at the time of intubation were intubated with a cuffed ETT (32.0%). Twelve infants were intubated with the Rüsch Super Safety ID 2.5 ETT, 21 with the Kimberly-Clarke Microcuff ID 3.0 ETT, and two infants who were intubated in the operating room, obtained the Rüsch Super Safety ID 3.5 ETT. Only one infant with a cuffed ETT and a bodyweight slightly below 3 kg developed post-extubation stridor, which resolved within 3 h after the administration of corticosteroids and nebulized epinephrine.

### Development of Subglottic Stenosis

In the study period, thirteen (=1, 2%) out of all infants requiring invasive mechanical ventilation within the study period (=1,098) developed a severe SGS at some point. Data regarding gestational age at birth, day of life and weight at first intubation as well as cause for intubation, day of life of SGS diagnosis, the use of cuffed and uncuffed ETTs before the diagnosis of SGS, the need for tracheostomy and other important comments are presented in Table 3. All infants suffering from acquired SGS underwent at least one intubation with an uncuffed ETT prior to the SGS diagnosis. 38.5% infants required laryngotracheal procedures, excluding tracheostomy. Approximately 60% of the infants with diagnosed SGS needed a tracheostomy.

**Table 3:** Characteristics of infants who developed a subglottic stenosis (SGS) needing a laryngeal intervention.

**Number**	**GA at birth [weeks]**	**DOL of 1st intubation**	**Weight at 1st intubation [kg]**	**Cause of 1st intubation**	**DOL of SGS diagnosis**	**Cuffed, uncuffed ETTs prior to SGS diagnosis**	**Need for tracheostomy**	**Comments**
1	26	92	2.7	ROP: Laser photocoagulation	131	2, 3	yes	1st intubation with cuffed ETT
2	23	3	0.59	RDS	87	0, 7	yes	
3	34	2	1.8	Tracheo-esophageal fistula	46	1, 4	no	Resection of subglottic narrowing
4	23	1	0.51	RDS	90	0, 1	no	Resection of subglottic stenosis on DOL 92 and 140
5	36	30	3.5	RSV bronchiolitis, CPR with difficult intubation	55	0, 1	yes	
6	23	1	0.53	RDS	84	0, 7	no	Separation of mucosal bridges
7	26	2	0.85	RDS	133	1, 4	yes	
8	38	2	0.79	Pulmonary hypertension	102	0, 3	yes	
9	34	5	1.8	Tracheo-esophageal fistula	151	0, 7	yes	
10	24	21	0.97	Sepsis	144	0, 1	yes	
11	42	3	3.3	Placement of a ventricular-peritoneal Shunt in Dandy-Walker Malformation	8	1, 1	no	Difficult first intubation, SGS resolved without surgical intervention
12	23	1	0.65	RDS	120	0, 3	yes	Laryngotracheal reconstruction at 10 month of age
13	24	1	0.66	RDS	46	0, 6	no	Cricotracheal mucosa resection at 9 month of age

^*^ All infants needed Larygo-/tracheocoscopy.

*GA, Gestational Age; DOL, days of life; RDS, Respiratory distress syndrome; ROP, Retinopathy of Prematurity; RSV, Rhino Syncytial Virus*.

## Discussion

In this retrospective cohort study, we report a low incidence (2.6%) of post-extubation stridor in infants with a body weight ranging from 2 to 6 kg. The incidence was not significantly elevated with cuffed ETTs compared to uncuffed ETTs (71.4 vs. 28.6%, *p* = 0.053). Reports about the incidence of post-extubation stridor vary. In children with an average age of 6.4 years (SD 4.5) undergoing anesthesia for diagnostic or surgical procedures, about 1.1% of children were found to suffer from post-extubation stridor, and 4.3% of those required re-intubation due to stridor (27). On the contrary, in a study investigating the incidence of stridor in infants with an average age of 1.0 month (range 0–201), stridor was identified in 18.7% of infants, among which the majority resolved with oxygen therapy or medication. But 10.7% of the infants presenting with stridor required re-intubation (28). However, previous intubation was not an exclusion criterion in this study and 70% of infants needed a tube exchange, which could explain the much higher percentage of stridor. With an increasing number of intubations, the risk for airway injury and stridor is higher. Three or more intubations have been associated with a higher risk for laryngeal injury in infants (29).

Our results show that cuffed ETTs were most often inserted in the operating room right before a surgical procedure (Table 1). This indicates that cuffed tubes are more frequently used by anesthesiologists than neonatologists, respectively. During our study period, the use of cuffed ETTs in general increased remarkably (Figure 2) which supports several reports showing that the percentage of anesthesiologists using cuffed ETTs in neonates has increased over the last 20 years (3, 4, 30–33). In our institution this may be attributed to the fact that from 2016 onward we agreed to use cuffed ETT whenever the infant with a bodyweight above 2 kg needed a surgical treatment. This change of the local policy was initiated due to the given advantages that come along with the use of cuffed ETTs during sensitive surgical procedures. These include decreased risk of aspiration (5, 6), possibility to adjust cuff volume (7), a smaller percentage of air leak (9, 11) and fewer tube replacements (8–10). Also, there was no given literature that the use of cuffed ETTs in children with a bodyweight below 3 kg come along with a higher incidence of post-extubation stridor. The largest prospective randomized trial by Weiss et al. (10) investigating stridor in 2,246 patients found a larger number of patients with stridor; however, they reported that in the cuffed group 4.4% developed stridor while in the uncuffed group this number amounted to 4.7%. They included children until the age of five and a bodyweight higher than three kilograms. Previous intubation was not an exclusion criterion. In the same study, the authors further reported that ETT exchange was far lower in the cuffed group (2.1 vs. 30.8%, *p* < 0.001) which since has been acknowledged as one of the major advantages of the cuffed ETTs. The authors also described minimal cuff pressure required to seal the trachea of about 10.6 (SD 4.3) cm H2O which is similar to our observations.

Although our results might indicate a higher risk of post-extubation stridor for cuffed ETTs compared to uncuffed ETTs, the small number of infants with stridor does not allow to draw any conclusion on the choice of ETT in order to lower the risk of post-extubation stridor in infants (OR 4.86, 95%-CI 0.93–25.56). However, two more aspects might argue an equivalent risk for post-extubation stridor between the cuffed vs. the uncuffed ETT: the more frequent administration of pre-extubation corticosteroids and the shorter duration of intubation in the cuffed group. The administration of pre-extubation corticosteroids was solely upon decision of the attending physician. Multivariate logistic regression identified the administration of corticosteroids before extubation to be the only significant association with post-extubation stridor (*p* < 0.001). In our policy, we consider corticoidsteroids prior to extubation whenever history of a difficult intubation in this infant is reported. In so far, the association of corticosteroid administration with post-exubation stridor might be referred to the fact that difficult intubation is prone to laryngeal injury. ETT air leak test was not performed, since its predictive power remains questionable (34).

Corticosteroids prior to extubation might reduce the incidence of stridor, particularly in high-risk patients. On the other hand, patients in the cuffed group were intubated for a shorter period than patients with an uncuffed ETT. Again, this observation might skew the OR in favor of the cuffed group since intubation is a known risk factor for the occurrence of stridor (15).

In the study by Veder et al. (28) cuffed ETTs have been identified as a predictor for post-extubation stridor in patients under the age of one. A recently conducted unblinded randomized controlled trial investigated the efficacy and safety of cuffed vs. uncuffed ETTs for infants of <3 months of age and found no difference in achieving an optimal ETT leak in the target range of 10–20% (35). The author reported that cuffed ETTs reduced re-intubations to optimize ETT size and episodes of atelectasis, and concluded that ETTs with cuff did not appear to increase post-extubation complications in infants with a bodyweight of more than 3 kg as opposed to the observational study by Velder et al. There is little evidence for the use of cuffed ETTs in infants below three kilograms, as cuffed ETTs are not recommended for this patient cohort (36). Thomas et al. retrospectively investigated a small cohort of infants with a bodyweight below three kilograms, who were intubated with a cuffed ETT for various reasons and compared them to infants who received an uncuffed ETT (46 patients in total). They found no significant difference in post-extubation stridor (37).

Subglottic stenosis represents one of the most severe forms of ETT-related injury, and the morbidity associated with it makes it an important issue (38). Traditionally, there are concerns relating the use of cuffed ETT and the risk of acquired SGS in infants and children (4). Hardly any infant in our study group developed severe SGS. This is in accordance with what has been reported recently by Greaney et al., who did not observe an increased risk of acquired SGS pediatric patients initially intubated with a low-pressure, high-volume cuff ETT (25). Most of the patients with acquired SGS had a history of prematurity and underwent several intubations, which has also been described by Greaney et al., (25). One infant in our cohort (Patient Nr.1 Table 3), who had been intubated with a cuffed ETT twice in the first place, developed SGS eventually. No stridor was described after the first extubation. This infant also received repeated intubations with uncuffed ETTs prior to the SGS diagnosis. Therefore, the origin of SGS remains unclear. Furthermore, Patient Nr.11 underwent intubation with a cuffed ETT in the first place. This initial intubation was described difficult and required several attempts until it succeeded. He presented with stridor after extubation and was re-intubated 12 h later because he became exhausted trying to breathe. Also, this intubation with an uncuffed ETT was reported difficult. In the laryngoscopy, a SGS was diagnosed which resolved within 2 weeks without the need for surgical intervention. This infant was the only one with post-extubation stridor (in the cuffed group) who developed a subglottic stenosis. However, because of the multiple intubation attempts the role of the cuffed ETT remains questionable for the development of the SGS.

### Limitations

Due to the retrospective character of the study, we must point out several limitations. Since all data was retrospectively extracted from PDMS, we had to rely on the accuracy of the data, which sometimes lacked precision. Due to a lack of definite stridor documentation, we decided to define post-extubation stridor every time an infant received stridor related medication (corticosteroids or nebulized epinephrine) within the first 6 h post- extubation, as the administration of drugs always must be documented. However, this implies that benign stridor that resolved without medication could not be identified in this study. Some authors argue that stridor is not an accurate marker for airway injury, because lesions have also been found post-extubation when performing endoscopy in the absence of stridor (39). Moreover, since the two groups were not randomized there might be an allocation bias.

Since we only included infants that were extubated at our NICUs, we did not include infants who were extubated in the operating room. Furthermore, a higher proportion of infants was intubated with a cuffed ETT in the OR, underwent a surgical procedure and were transferred to the NICU with an ETT *in-situ*. These factors are also potential cofounders.

Our cohort includes infants with complex illnesses that need admission to a NICU either after surgery, or because of the illness itself. These critically ill infants might be in general at a higher risk for stridor than other infants. Hence, our results may not be generalized to other patient groups.

We did not screen for difficult intubation (e.g., more than one attempt) as there is no extensive documentation in our PDMS in this regard. Unsuccessful attempts of intubation might increase the risk for upper airway injuries, which could have influenced our results.

Furthermore, the present study might be under powered to prove “no association” between the use of cuffed ETTs and pot-extubation stridor. It does support results from the literature, but is not conclusive.

A strength of our study is the large study cohort. Moreover, we studied a cohort of patients that had been admitted to one of our NICUs and did not solely focus on intubation for pediatric anesthesia. We further considered only the first intubation in order to reject the impact of preceding endotracheal intubations on post-extubation complications.

## Conclusion

The incidence of post-extubation stridor is low in infants with a body weight ranging from 2 kg to 6 kg and without multiple periods of intubation. This study supports recent findings that the use of cuffed ETTs with appropriately adjusted cuff pressure does not come along with a higher incidence of post-extubation stridor in infants with a body weight between 2 and 6 kg However, further research is needed to confirm this observation.

## Contribution to the Field Statement

There is a lack of evidence that the use of cuffed ETTs in infants, even below a bodyweight of 3 kg, correlates with a higher risk for post-extubation stridor or the development of subglottic stenosis. In this retrospective study, we included data of the first intubation of all infants with a bodyweight between 2 and 6 kg admitted to the NICU who required invasive mechanical ventilation. Our analysis showed a low incidence of post-extubation stridor in the total, with a slightly higher incidence of stridor in infants intubated with a cuffed ETT. However, further analysis in this cohort showed that the use of a cuffed ETT does not come along with a higher risk for acquired subglottic stenosis needing surgical intervention. This study underlines that the use of cuffed ETTs in infants is safe, even in patients with a bodyweight between 2 and 3 kg. It provides feasible data to support the statement that widely spread concerns about the use of cuffed ETTs in infants may not be justified.

## Data Availability Statement

The original contributions presented in the study are included in the article/supplementary materials, further inquiries can be directed to the corresponding author/s.

## Ethics Statement

The studies involving human participants were reviewed and approved by Institutional Review Board of the Medical University of Vienna (N. 1581/2019). Written informed consent from the participants' legal guardian/next of kin was not required to participate in this study in accordance with the national legislation and the institutional requirements. Written informed consent was not obtained from the minor(s)' legal guardian/next of kin for the publication of any potentially identifiable images or data included in this article.

## Author Contributions

KB, LP, and TW contributed to conception and design, drafted the initial study protocol, collected and analyzed data, drafted the article, and reviewed and revised the manuscript. LP and EK substantially contributed to analysis and interpretation of data and revising the manuscript critically. IR, MW, HM, and AB helped with analyzing, and critically reviewed and revised the manuscript for important intellectual content. All authors approved the final manuscript as submitted and agreed to be accountable for all aspects of the work.

## Conflict of Interest

The authors declare that the research was conducted in the absence of any commercial or financial relationships that could be construed as a potential conflict of interest.

## Publisher's Note

All claims expressed in this article are solely those of the authors and do not necessarily represent those of their affiliated organizations, or those of the publisher, the editors and the reviewers. Any product that may be evaluated in this article, or claim that may be made by its manufacturer, is not guaranteed or endorsed by the publisher.
